# Physiological performance of glyphosate and imazamox mixtures on *Amaranthus palmeri* sensitive and resistant to glyphosate

**DOI:** 10.1038/s41598-019-54642-9

**Published:** 2019-12-03

**Authors:** Manuel Fernández-Escalada, Ainhoa Zulet-González, Miriam Gil-Monreal, Mercedes Royuela, Ana Zabalza

**Affiliations:** Institute for Multidisciplinary Research in Applied Biology (IMAB), Universidad Pública de Navarra, Campus Arrosadia s/n, 31006 Pamplona, Spain

**Keywords:** Plant cell biology, Plant physiology

## Abstract

The herbicides glyphosate and imazamox inhibit the biosynthetic pathway of aromatic amino acids (AAA) and branched-chain amino acids (BCAA), respectively. Both herbicides share several physiological effects in the processes triggered in plants after herbicide application that kills the plant, and mixtures of both herbicides are being used. The aim of this study was to evaluate the physiological effects in the mixture of glyphosate and imazamox in glyphosate-sensitive (GS) and -resistant (GR) populations of the troublesome weed *Amaranthus palmeri*. The changes detected in the physiological parameters after herbicide mixtures application were similar and even less to the changes detected after individual treatments. This pattern was detected in shikimate, amino acid and carbohydrate content, and it was independent of the *EPSPS* copy number, as it was detected in both populations. In the case of the transcriptional pattern of the AAA pathway after glyphosate, interesting and contrary interactions with imazamox treatment were detected for both populations; enhancement of the effect in the GS population and alleviation in the GR population. At the transcriptional level, no cross regulation between AAA and BCAA inhibitors was confirmed. This study suggests that mixtures are equally or less toxic than herbicides alone, and would implicate careful considerations when applying the herbicide mixtures.

## Introduction

**Figure 1:**
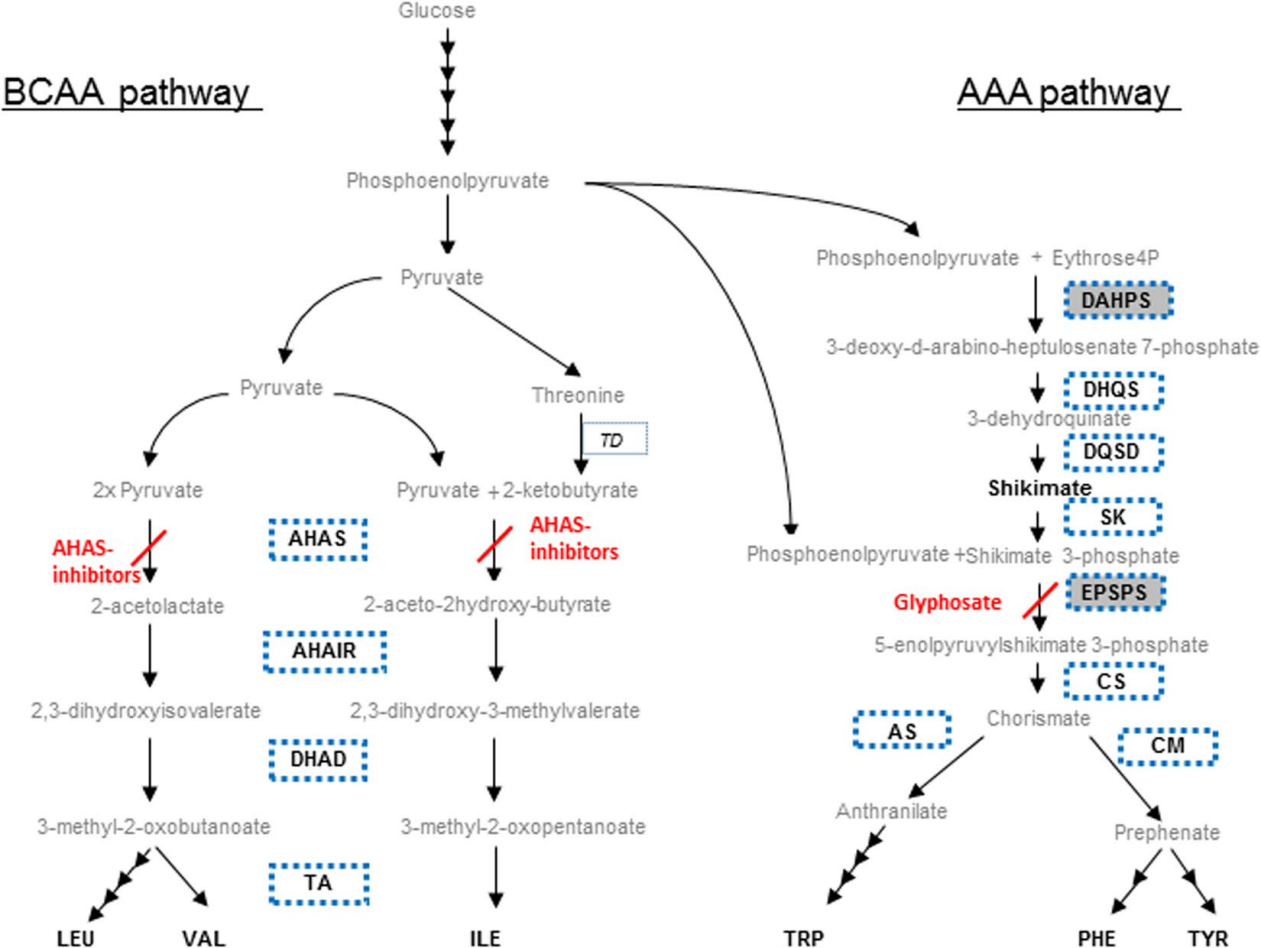
Aromatic amino acid pathway (AAA pathway) (in the right side) with their main enzymes: D-arabino- heptulosonate 7-phosphate synthase (DAHPS), dehydroquinate synthase (DHQS), 3-dehydroquinate dehydratase/shikimate dehydrogenase (DQSD), shikimate kinase (SK), 5-enolpyruvylshikimate 3-phosphate synthase (EPSPS), chorismate synthase (CS), chorismate mutase (CM) and anthranilate synthase (AS). Branched-chain amino acid pathway (BCAA pathway) (in the left side) with their main enzymes: Acetohydroxy acid synthase (AHAS), acetohydroxyacid isomer reductase (AHAIR), dihydroxyacid dehydratase (DHAD) and BCAA transaminase (TA). Threonine deaminase (TD) is the enzyme that synthesizes 2-ketobutyrate, needed to start the reactions in the branch of Isoleucine (ILE) biosynthesis. The source of carbon for both pathways is phosphoenolpyruvate (PEP) that comes from the photosynthesized glucose through glycolysis. Final products are leucine (LEU), valine (VAL), and isoleucine (ILE) in BCAA pathway, and tryptophan (TRP), phenylalanine (PHE) and (TYR) tyrosine in AAA pathway. *Data included in this study:* the expression level of the enzymes represented in bold letters inside a dotted blue box, the protein amount of the enzymes inside a grey box and the content of metabolites marked in bold black letters. Other intermediate products are represented in grey lower case letters.

The aromatic amino acid (AAA) biosynthesis pathway (Fig. 1) transforms the inputs of carbon into the essential amino acids phenylalanine (Phe), tyrosine (Tyr) and tryptophan (Trp)^1^ with several successive enzymatic reactions. These AAAs are used by the plant to form proteins; in addition, they are precursors for secondary metabolites, and some of them are involved in plant stress tolerance^2,3^. The target of the herbicide glyphosate is the 5-enolpyruvylshikimate-3-phosphate synthase (EPSPS) enzyme^4^ of the AAA pathway, which makes this step greatly important from an agronomic management standpoint.

The branched chain amino acid (BCAA) biosynthesis pathway (Fig. 1) leads to the formation of valine (Val), leucine (Leu) and isoleucine (Ile)^5^. Acetohydroxy acid synthase (AHAS) has a key position in the pathway, since the enzyme catalyzes not only the synthesis of acetolactate, the valine and leucine precursor from pyruvate, but also that of acetohydroxybutyrate, the isoleucine precursor from alpha-ketobutyrate and pyruvate^6^. Due to its relevance in the BCAA biosynthesis pathway, AHAS has been widely used as target point for herbicides^7^. There are five different chemical classes of AHAS-inhibitors: sulfonylureas, imidazolinones, triazolopyrimidines, sulfonylaminocarbonyl triazolinones and pyrimidinyl-oxy-benzoates^8^.

Although the target enzymes of the herbicides in the BCAA and AAA biosynthesis pathways are known, it is still unclear how exactly the inactivation of AHAS or EPSPS results in plant death. Previous findings showed that both AHAS and EPSPS inhibitors cause growth arrest followed by a slow plant death of the herbicide-treated plants^9,10^. Both types of herbicides provoke an accumulation of free amino acids^11–15^, a decrease in the soluble protein content^12,16,17^ and accumulation of carbohydrates^11,12,15,18^. Although they target different enzymes located in different pathways, these common physiological effects suggest that these herbicides kill plants by similar mechanisms.

The hypothesis of the existence of cross regulation of amino acid metabolic pathways has been proposed^19–21^ and several studies suggest a cross relationship between AAA and BCAA biosynthesis pathways. The contents of many minor amino acids vary in concert with different amino acid biosynthetic families^22^, and the closest correlation in these variations occurs between AAA and BCAA^13,22^. Moreover, some specific interactions between AHAS-inhibitors involved in Leu synthesis and the levels of Tyr and Phe have been described^23^.

The repeated use of glyphosate and AHAS-inhibitors selects for the corresponding resistances in weed populations^24^. Now there are 159 weed species with at least one population with resistance to AHAS-inhibitors^25,26^. The most important cause of resistance is the mutations in the AHAS protein^8^. To date, 41 weed species with at least one population with resistance to glyphosate have been reported. One of the most problematic weed species resistant to glyphosate is *Amaranthus palmeri* S. Wats^8,27^. Although non-target-site resistance mechanisms to glyphosate have been described recently^28^, the most common resistance mechanism to glyphosate is the target-site amplification of the *EPSPS* gene^29–31^, as has been described in several species^32,33^. When this gene is overexpressed, the EPSPS enzyme accumulates so that the recommended field dose of glyphosate is not sufficient to inhibit EPSPS activity and consequently the plants survive.

One of the most used practices to control glyphosate-resistant weeds is to mix glyphosate with AHAS-inhibitors^34–39^. Herbicide mixtures can interact in three different ways (antagonistically, additively, and synergistically)^40^ and there is a critical knowledge gap in the evaluation of AHAS-inhibitors and glyphosate mixtures as previous studies showed no conclusive results. In addition, since both herbicides induce an increase in the content of free amino acids (as a consequence of the inhibition of different pathways), and a cross regulation in the amino acid metabolic pathways might exist, the use of both herbicides on the same plant could lead to a slighter (or at least different) response than the sum of the responses to individual herbicides.

The use of effective mixtures is important for reducing selection pressure on individual target-sites, which causes the selection of favorable resistance mechanisms (such as mutations) to tolerate the herbicide and the appearance of resistances. In the case of *A. palmeri*, it is of particular importance because populations of this species have developed multiple resistances meaning that they are not only resistant to glyphosate but also to AHAS-inhibitors^41,42^.

The full comprehension of the physiology of individual and mixture herbicidal effects might enable knowledge-based adoption of alternative weed management practices in order to control the evolution of resistant weeds. Indeed, both synergism^43^ and antagonism^44^ have been described after mixing other herbicides. The close relationship between AAA and BCAA biosynthetic pathways and the common physiological effects provoked by EPSPS and AHAS-inhibitors (as they have been introduced above) makes interesting to study the physiological effects of their mixtures in plants. If synergism is detected as the main interaction, this could have agronomic implications such as the possibility of lowering application rates in these herbicide mixtures. On the contrary, antagonism would implicate the application of the full recommended rate of each herbicide to weed control.

The main objective in this study was to evaluate the physiological effects of mixing glyphosate with the AHAS-inhibitor imazamox and if the effect was different if the treated plant was resistant to glyphosate. To this end, the effects of both herbicides alone and their mixtures on known physiological markers were evaluated (shikimate, free amino acids and carbohydrates) on two populations of *A. palmeri* that were sensitive (GS) or resistant (GR) to glyphosate. Additionally, in order to clarify the global regulatory mechanisms of the AAA pathway and if there is a cross regulation between AAA and BCAA biosynthetic pathways, the relative expression of the genes of AAA and BCAA pathways based on *m*RNA levels and the protein content of key enzymes in the AAA pathway were tested.

## Materials and Methods

### Plant material and treatment application

Seeds from the two biotypes of *A. palmeri*, i.e., sensitive (GS) and resistant (GR) to glyphosate, were originally collected from North Carolina (USA)^11,30^. The resistance mechanism of the GR biotype is *EPSPS* gene amplification^30^, with 47.5 more gene copies in GR than in GS plants^11^. Germination and plant growth were performed according to procedures described earlier^11^. Briefly, after germination the seeds were transferred to aerated 2.7 L hydroponic tanks in a phytotron (day/night, 16 h/8 h; light intensity, 500 μmol s^−1^ m^−2^ PAR; temperature, 22/18 °C; relative humidity of the air, 60/70%). Throughout the course of the experiment, the plants remained in the vegetative phenological stage. All treatments were applied to three week-old plants after selecting individuals of similar size and vigor. Glyphosate, (commercial formula, Glyfos, BayerGarden, Valencia, Spain) was applied at both 0.25 times recommended field rate (0.25 G = 0.21 kg ha^−1^) and at the recommended field rate (1 G = 0.84 kg ha^−1^)^27^. The AHAS inhibitor imazamox (commercial formula, 4% P/V (Pulsar 40®, BASF, Barcelona, Spain)) was applied at the sublethal dose of 1.5 mg active ingredient L^−1^ ^45^ after conducting preliminary dose-responses studies to select imazamox dose. The mixtures of imazamox with the two doses of glyphosate were also applied (0.25 G + I and 1 G + I). Glyphosate treatment was performed using an aerograph (Definik; Sagola, Vitoria-Gasteiz, Spain) connected to a compressor (Werther one, Breverrato) with the following settings: 60 W; 10 L m^−1^; 2.5 bar at a rate of 500 L ha^−1^. Imazamox was added and mixed to the nutrient solution where it remained the whole time of the experiment. Another set of plants sprayed with water was used as the control reference. The leaves of both populations of *A. palmeri* were collected 3 days after treatment, ground to a fine powder under liquid N_2_ using a mixer mill as previously described^11^.

### Quantitative reverse transcription-PCR

The relative transcript level was measured for all genes of the AAA synthesis pathway, corresponding to eight enzymes; and four genes of the BCAA synthesis pathway. RNA extraction and the subsequent cDNA extraction were performed as described in Fernandez-Escalada^46^. Quantitative RT-PCR (qRT-PCR) was conducted by using techniques and primers detailed before^46^. Relative transcript levels were calculated as E_GOI_^CP^_GOI_^control−CP^_GOI_^treated^/E_REF_^CP^_REF_^control−CP^_REF_^treated^ ^46^, where the control of GS was used to calculate all GS values and the control of GR was used to calculate all GR values.

### EPSPS and DAHPS immunoblotting

Protein extraction and immunoblotting were performed according to standard techniques and as described previously^46^. In the case of EPSPS, the protein amount loaded per well in each population was different and is specified in the figure legends. Membrane signals were normalized according to total soluble protein loading quantity.

### Shikimate content determination

Three leaf disks (4 mm diameter) were excised from the youngest leaf of each plant for shikimate content determination. Leaf disks were placed in 2 mL Eppendorf tubes and stored at −80 °C until analysis. Shikimate was extracted as described previously^47^. Shikimate content was quantified spectrophotometrically^48^.

### Amino acid content determination

Ground leaf samples (0.1 g) were homogenized in 1 M HCl for amino acid extraction. Protein precipitation was performed after incubation on ice and centrifugation^13^. After derivatization with fluorescein isothiocyanate, the amino acid content was measured by capillary electrophoresis coupled to a laser-induced fluorescence detector, as described before^13^. Analyses were performed at 20 °C and at a voltage of +30 kV.

### Carbohydrate content determination

The total soluble sugar (glucose, fructose, and sucrose) content (TSS content) was determined in ethanol-soluble extracts, and the ethanol-insoluble residue was extracted for starch analysis as in Zabalza *et al*.^49^. The starch and TSS contents were determined by ion chromatography (930 Compact IC Flex, Metrohm AG Ionenstrasse CH-9100 Herisau, Switzerland), following the manufacturer’s instructions (Gomensoro Scientific Instrumentation, Madrid, Spain). The sample dilutions used for soluble carbohydrates and starch were 1:10 and 1:50, respectively. To prepare the samples, the eluent used was 300 mM NaOH/1 mM sodic acetate in Mili Q water solution. The applied current was 200–500 mA, with pressure of 1000–1200 psi and temperature between 30 and 35 °C.

### Statistical analysis

All analyses were performed using four biological replicates from two independent experiments. For all parameters tested, the difference between untreated plants of each population was evaluated using Student’s *t-*test, which found no significant differences; thus, this result is not mentioned in the text.

Principal component analysis (PCA) was performed on the data sets obtained from expression and metabolite content with the MetaboAnalyst data annotation approach^50^ (http://www.metaboanalyst.ca/MetaboAnalyst/faces/Home.jsp). The Heatmap option, implemented in the MetaboAnalyst tool was used. The integrity of the data was checked. The data was autoscaled and normalized (mean-centered and divided by the standard deviation of each variable).

For each population, data for each parameter was subjected to two-way analysis of variance (ANOVA; p < 0.05). Factors used in the analysis were imazamox and glyphosate. Before ANOVA, data were checked for normality and the homogeneity of variances, and log-transformed to correct deviations from these assumptions when needed. Post-hoc comparisons were tested using the Bonferroni *post-hoc* test at a significance level of p < 0.05). For each population, significant differences are highlighted in the figures using different letters.

Statistical analyses were performed using IBM SPSS statistics 24.0 (IBM, Corp., Armonk, NY, United States).

## Results

To evaluate whether the physiological effects provoked by glyphosate and imazamox mixtures were different to the ones provoked by herbicides alone, GS and GR *A. palmeri* populations were treated with two doses of glyphosate (0.25 G and 1 G), imazamox (I) or their mixtures (0.25 G + I and 1 G + I).

Leaf samples were taken after three days, before obvious symptoms were observed, as it is shown in Supplementary Fig. 1. Aspect of the plants showed that GS plants treated with the 1 G + I mixture were more affected, but they were still green and turgid enough. This time point was chosen in order to evaluate physiological and biochemical plant responses induced by the herbicide but not directly resulting from cell death. The evaluation of such responses focused on the AAA and BCAA biosynthetic pathways, which are schematized in Fig. 1. Additionally, measured metabolites and genes are specified in Fig. 1.

The dataset of measured parameters was treated using Principal Component Analysis (PCA) to extract the parameters that are most important in assessing variation after herbicide treatment. It was assessed the interaction between glyphosate and imazamox in each population for all the parameters tested (Supplementary Table 1). Out of the 28 parameters evaluated, it was detected interaction in 7 and 20 parameters in GS and GR population, respectively. The study of the effect of the treatments on each population was approached by analyzing gene expression and metabolite contents as two separate datasets.

### Biosynthetic pathways of aromatic and branched chain amino acids: Gene expression level and protein content

**Figure 2:**
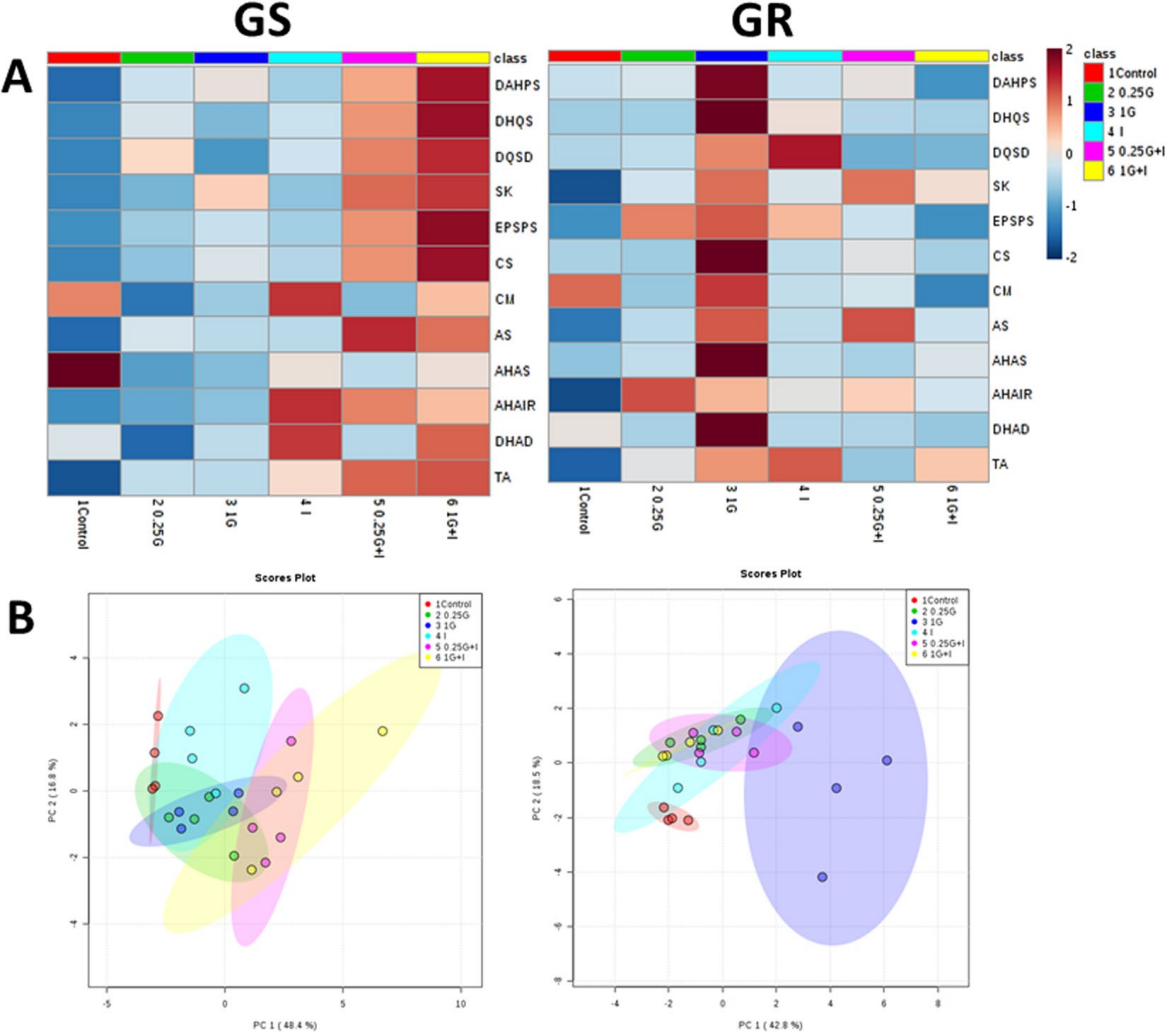
Overview of the expression profiles of the genes of the enzymes of aromatic amino acid (AAA) and branched-chain amino acid (BCAA). (**A**) Heatmap visualization of the expression profile in GS (left) and GR (right) populations. Each column represents the group average. Values were centred to the mean of the respective mean and scaled to unit variance. White colour shows values close to the mean and red- and blue- coloured values are higher and lower, respectively, than the mean. Enzymes of the AAA pathway: D-arabino- heptulosonate 7-phosphate synthase (DAHPS), dehydroquinate synthase (DHQS), 3-dehydroquinate dehydratase/shikimate dehydrogenase (DQSD), shikimate kinase (SK), 5-enolpyruvylshikimate 3-phosphate synthase (EPSPS), chorismate synthase (CS), chorismate mutase (CM) and anthranilate synthase (AS). Enzymes of the branched-chain amino acid (BCAA) pathway: acetohydroxy acid synthase (AHAS), acetohydroxyacid isomer reductase (AHAIR), dihydroxyacid dehydratase (DHAD) and BCAA transaminase (TA). Samples were taken 3 days after herbicide treatment. Untreated plants were sprayed with water (Control; C). Plants were treated with 0.21 kg ha^−1^ of glyphosate (0.25 G), 0.84 kg ha^−1^ of glyphosate (1 G), 1.5 mg L^−1^ of imazamox (I) or their mixtures (0.25 G + I and 1 G + I). (**B**) Principal component analysis (PCA) of expression level of AAA and BCAA biosynthetic pathways in GS (left) and GR (right) populations. The PCA is presented as the best combination of the first five dimensions. Each data point represents an independent sample. The first two axes of the PCA explain 65% and 61% of the variance in GS and GR populations, respectively.

**Figure 3:**
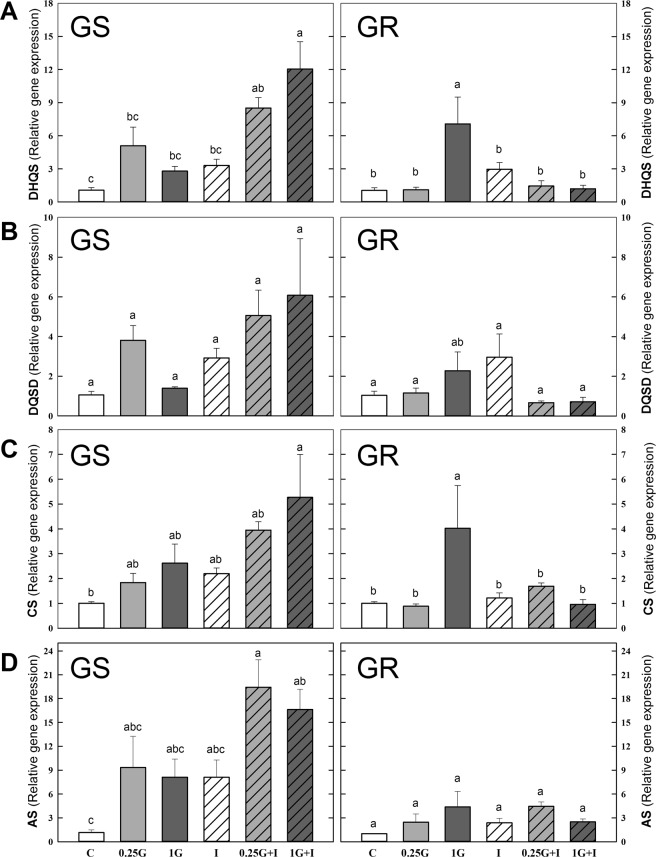
Transcript abundance of genes in aromatic amino acid (AAA) pathway enzymes. Relative transcript abundance was normalized using the normalization gene *beta tubulin* and each population to its own control in *Amaranthus palmeri* plants 3 days after herbicide treatment in sensitive (GS) and resistant (GR) populations of: (**A**) Dehydroquinate synthase (DHQS), (**B**) 3-dehydroquinate dehydratase/shikimate dehydrogenase (DQSD), (**C**) chorismate synthase (CS) and (**D**) anthranilate synthase (AS). Untreated plants were sprayed with water (Control; C). Plants were treated with 0.21 kg ha^−1^ of glyphosate (0.25 G), 0.84 kg ha^−1^ of glyphosate (1 G), 1.5 mg L^−1^ of imazamox (I) or their mixtures (0.25 G + I and 1 G + I). (Mean ± SE; n = 4). In each graph, different letters refer to statistically significant differences between treatments (two-way ANOVA followed by the Bonferroni *post hoc* test (P < 0.05)).

**Figure 4:**
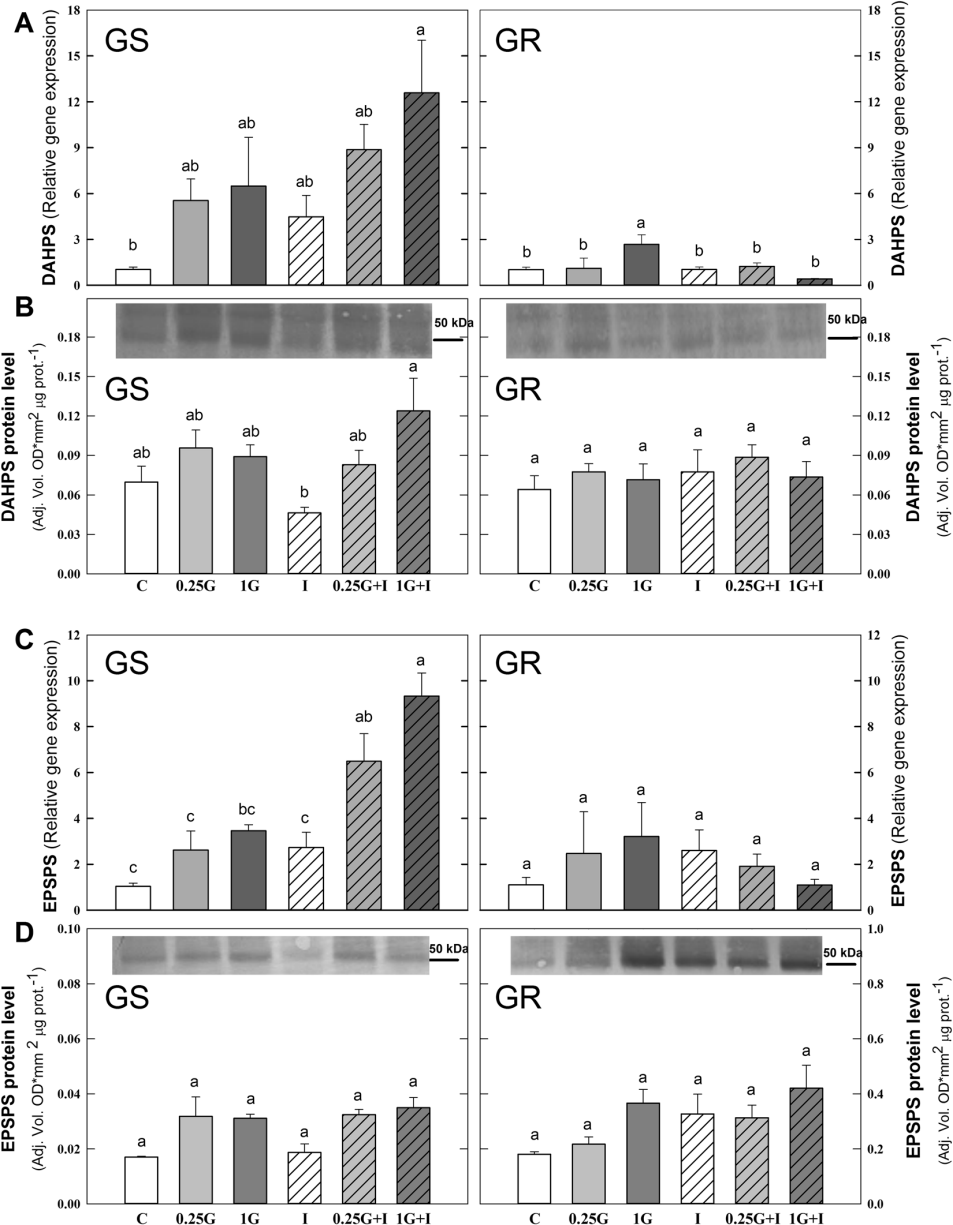
DAHPS and EPSPS: expression and protein levels in glyphosate sensitive (GS) and resistant (GR) *Amaranthus palmeri* populations. Transcript abundance in D-arabino-heptulosonate 7-phosphate synthase (DAHPS; **A**) and EPSPS 5-enolypyruvylshikimate 3-phosphate synthase (EPSPS; **C**) genes. Relative transcript abundance was normalized using the normalization gene *beta tubulin* and each population to its own control. Normalized protein level of DAHPS (**B**) an EPSPS (**D**). Normalization of the intensity of the bands expressed as optical density for unit of area per μg of protein. Total soluble protein (40 μg per well in B, 80 μg per well in D (GS) and 15 μg per well in D (GR)) were fractioned by 12.5% SDS-PAGE and blotted. In all cases results are accompanied with a representative picture of the immunoblot. Full-length blots are presented in Supplementary Figs. 2 and 3. Samples were taken 3 days after herbicide treatment. Untreated plants were sprayed with water (Control; C). Plants were treated with 0.21 kg ha^−1^ of glyphosate (0.25 G), 0.84 kg ha^−1^ of glyphosate (1 G), 1.5 mg L^−1^ of imazamox (I) or their mixtures (0.25 G + I and 1 G + I). (Mean ± SE; n = 4). In each graph, different letters refer to statistically significant differences between treatments (two-way ANOVA followed by the Bonferroni *post hoc* test (P < 0.05)).

The expression pattern of the genes of the AAA and BCAA pathways in the leaves of both populations was affected by herbicide treatments (Fig. 2A). The values were subjected to a Principal Component Analysis (PCA) and converted to a set of two PCs that contributed 65% and 61% of the total variance, in GS and GR population respectively (Fig. 2B). With these percentages, a separation in PC1 was observed between 1G and the other treatments in GR population. Next, it was performed an evaluation of the loadings, the parameters (in this case the expression level) that exhibited the greatest influence on the discrimination. For PC1 and PC2, and in both populations, the top loadings included expression of the following genes of the AAA pathway: *DAHPS* (D-arabino- heptulosonate 7-phosphate synthase)*, DHQS* (dehydroquinate synthase)*, DQSD (*3-dehydroquinate dehydratase/shikimate dehydrogenase), *EPSPS, CS* (chorismate synthase) and *CM* (chorismate mutase). Figures 3 and 4 (A and C) show the mean separation by individual two way ANOVAs between the treatments in both populations for these most significant genes. The effect of each treatment on all genes of AAA and BCAA pathways are included in the Supplementary Table 2.

In the GR population, as detected by PCA analysis and visualized in Figs. 1G and 2 treatment caused general increase in the expression of AAA genes, that was significant in the case of *DAHPS* (Fig. 4A), *DHQS*, and *CS* (Fig. 3) and. Imazamox applied alone did not induce any general pattern in the relative expression of the genes of the AAA pathway in any of the populations (Figs. 3 and 4). Interestingly, an opposite pattern in the relative gene expression in response to mixtures was detected between the populations. The detected general induction of the expression of the AAA pathway in the GS population after mixtures treatments (Figs. 2–4) was higher than the pattern after glyphosate alone, being significant in most cases. On the contrary, in the GR population, there was no induction in the AAA pathway enzyme transcripts with mixtures (Figs. 2–4). The increased expression detected after 1 G in GR population was abolished in the mixtures and leaves of 1 G + I treatment showed expression levels similar to control values (Figs. 2, 3 and 4A).

The expression pattern of the genes involved in the BCAA pathway was also studied (Fig. 2, Supplementary Tables 1 and 2). When glyphosate was applied alone, there were no significant changes detected in any of the populations and no significant or general changes in BCAA gene expression were detected after the inhibition of one of the enzymes of the BCAA pathway, AHAS, by imazamox alone. In the same way, the herbicide mixtures did not induce any remarkable pattern in the relative expression of the enzymes in this pathway.

The protein contents of two important proteins of AAA pathway (DAHPS and EPSPS) were measured (Fig. 4B,D, Supplementary Table 1). The only significant change in the protein content was detected in DAHPS in the GS population, where 1 G + I treatment provoked a significant increase of the DAHPS protein with respect to I (Fig. 4B). Both populations showed no effect in response to imazamox alone, and there were no significant differences between glyphosate treatments and their mixtures with imazamox (Fig. 4B). The EPSPS protein content in non-treated plants was 10-fold higher in the *A. palmeri* GR population than in the GS population (Fig. 4D). EPSPS protein content was not significantly affected by any of the treatments in any of the populations (Fig. 4D).

**Figure 5:**
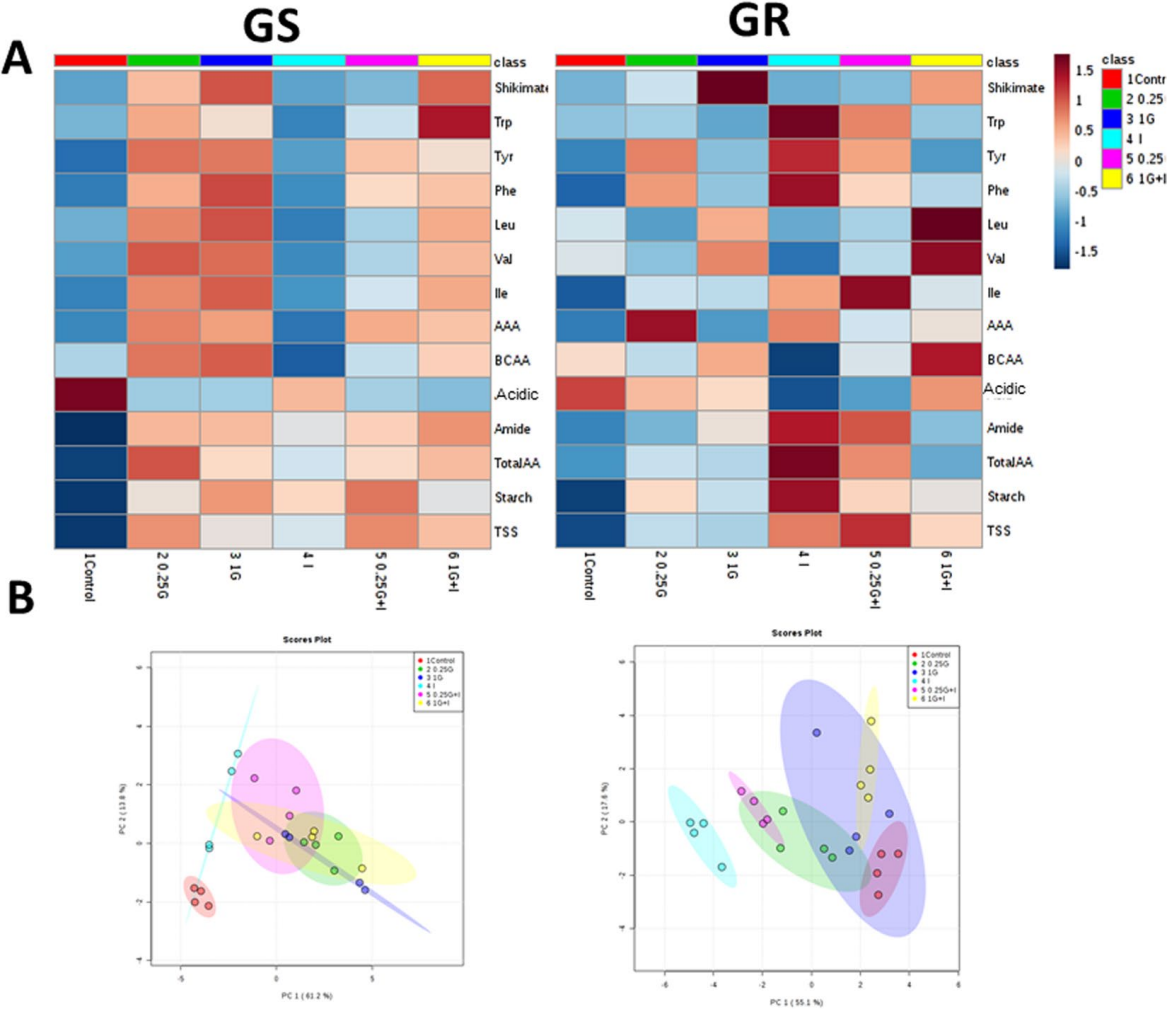
Overview of metabolite content: shikimate, amino acids and carbohydrates. (**A)** Heatmap visualization of the metabolite content of GS (left) and GR (right) populations. Each column represents the group average. Values were centred to the mean of the respective mean and scaled to unit variance. White colour shows values close to the mean and red- and blue- coloured values are higher and lower, respectively, than the mean. Shikimate content. Aromatic amino acid (AAA) content: tryptophan (Trp), tyrosine (Tyr), phenylalanine (Phe) and AAA percentage of the total free amino acid pool (AAA). Branched chain amino acid content (BCAA): leucine (Leu), valine (Val), phenylalanine (Ile) and BCAA percentage of the total free amino acid. Total free amino acid content, acidic amino acids (glutamic acid (Glu) + aspartate (Asp)) as percentage of the total free amino acid pool and amide amino acids (glutamine (Gln) + asparagine (Asn)). Total soluble sugar (fructose, glucose and sucrose) content (TSS) and starch. Samples were taken 3 days after herbicide treatment. Untreated plants were sprayed with water (Control; C). Plants were treated with 0.21 kg ha^−1^ of glyphosate (0.25 G), 0.84 kg ha^−1^ of glyphosate (1 G), 1.5 mg L^−1^ of imazamox (I) or their mixtures (0.25 G + I and 1 G + I). (**B**) Principal component analysis (PCA) of metabolite profile of shikimate, amino acids and carbohydrates in GS (left) and GR (right) populations. The PCA is presented as the best combination of the first five dimensions. Each data point represents an independent sample. The first two axes of the PCA explain 75% and 73% of the variance in GS and GR populations, respectively.

### Metabolite profile of GS and GR populations after herbicide treatments

The effect of the treatments on amino acid profile, shikimate and carbohydrate content was evaluated (Fig. 5A). These metabolites were selected on the basis that they are known physiological markers of glyphosate and/or ALS inhibitors. In each population, PCA analysis was performed to classify and characterize herbicide treatments based on metabolite contents (Fig. 5A). PC1 and PC2 explained about 75% in GS and about 73% in GR. Notably, in both populations there was a treatment relatively easy to discriminate from the others: control in the case of GS and imazamox in the case of GR population. After identifying factor loadings in both populations, the parameters more significant were selected and they are shown in Figs. 5 and 7. Individual ANOVAs comparing the effect of all treatments on all metabolites are included in Supplementary Table 3 (data of GS population and Supplementary Table 4 (data of GR population).

**Figure 6:**
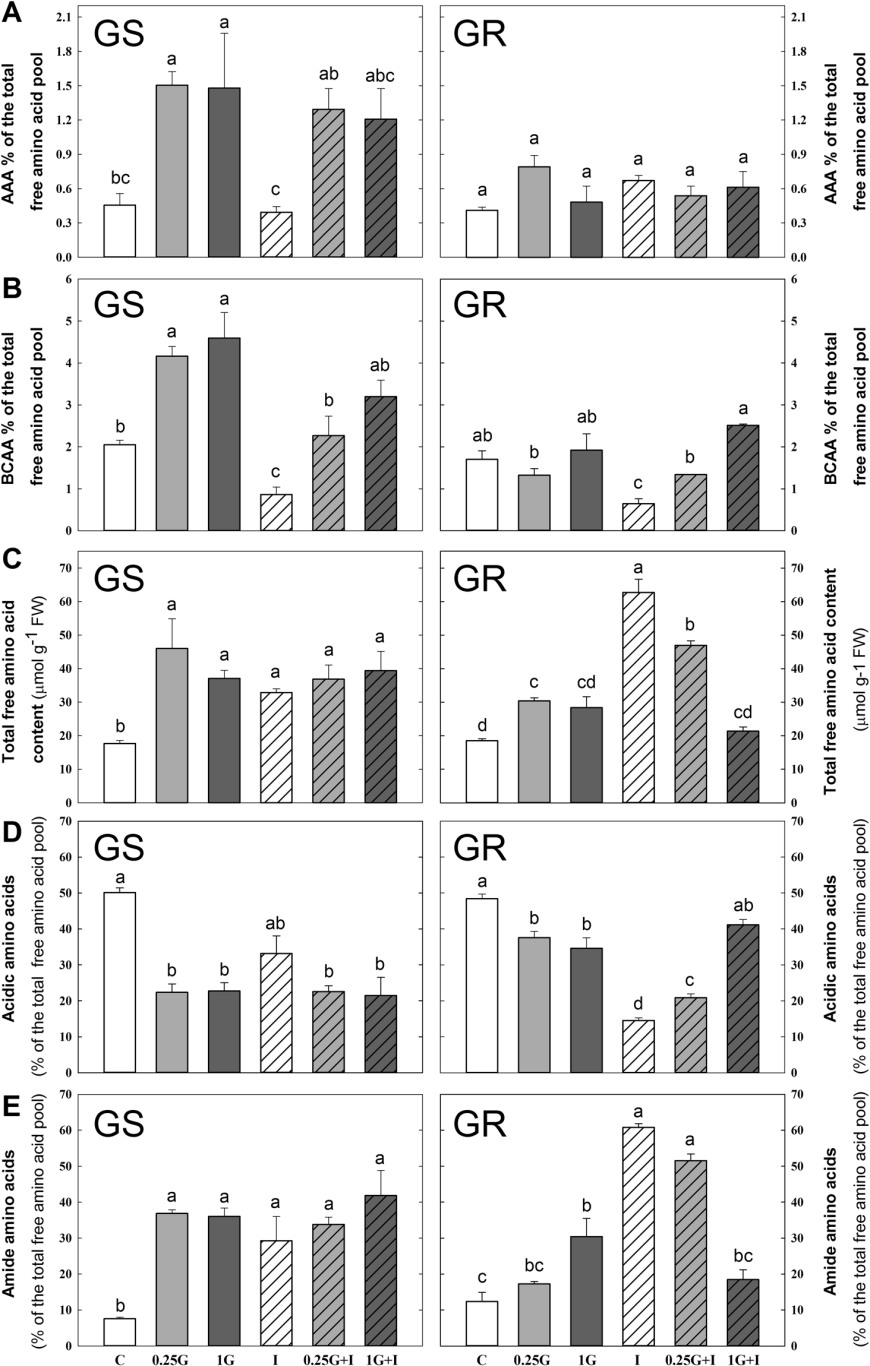
Amino acid profile. Aromatic amino acid (AAA; **A**) and branched chain amino acid (BCAA, **B**) percentages of the total free amino acid, total free (**C**), acidic (**D**) and amide (**E**) amino acid content as percentage of the total free amino acid pool in the leaves of glyphosate sensitive (GS) and resistant (GR) *Amaranthus palmeri* plants 3 days after herbicide treatment, measured by capillary electrophoresis in leaf acidic extracts. Untreated plants were sprayed with water (Control; C). Plants were treated with 0.21 kg ha^−1^ of glyphosate (0.25 G), 0.84 kg ha^−1^ of glyphosate (1 G), 1.5 mg L^−1^ of imazamox (I) or their mixtures (0.25 G + I and 1 G + I). (Mean ± SE; n = 4). In each graph, different letters refer to statistically significant differences between treatments (two-way ANOVA followed by the Bonferroni *post hoc* test (P < 0.05)).

**Figure 7:**
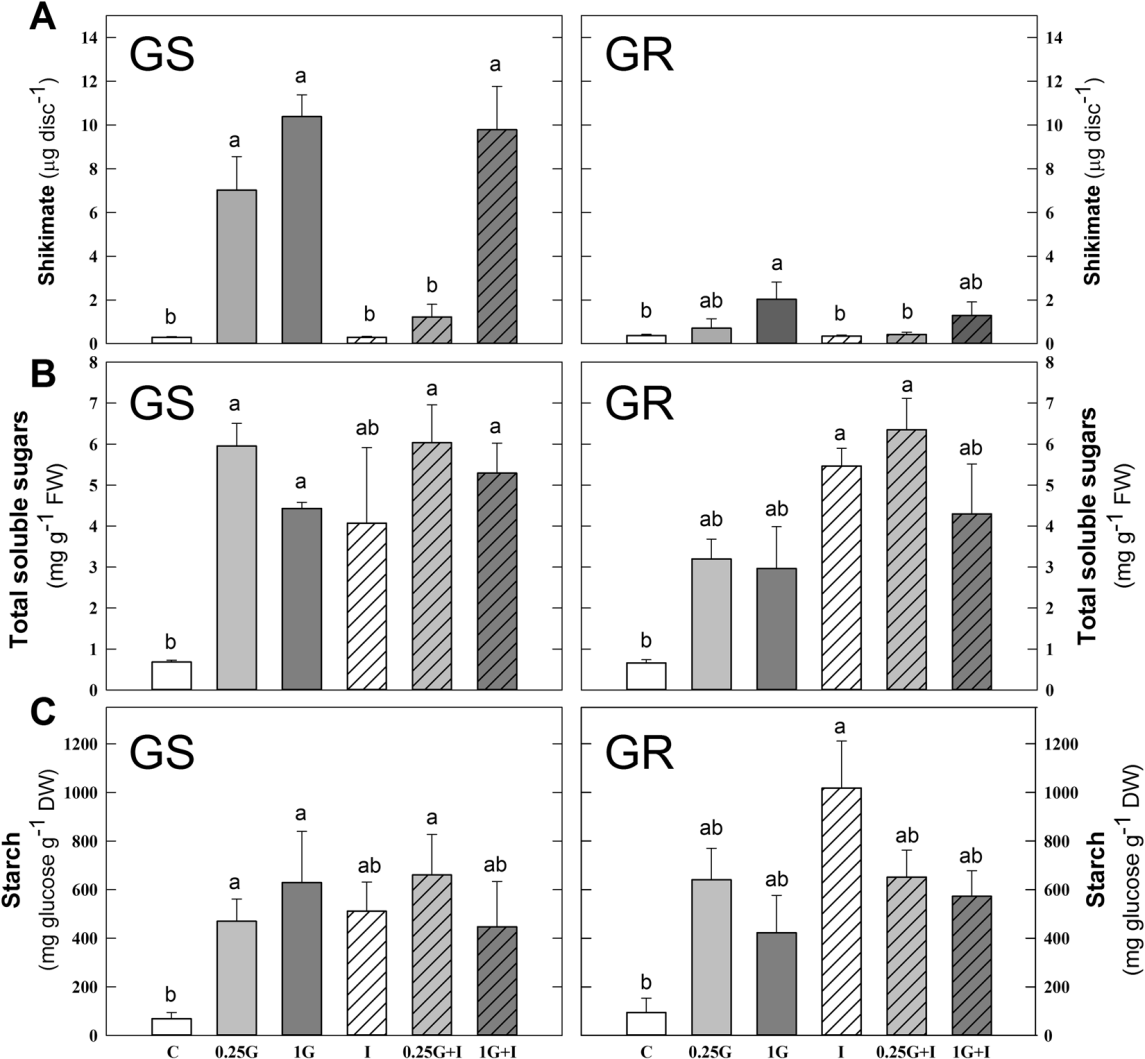
Shikimate and carbohydrate content. Shikimate (**A**) total soluble sugar (fructose, glucose and sucrose) (**B**) and starch (**C**) contents in in the leaves of glyphosate sensitive (GS) and resistant (GR) populations of *Amaranthus palmeri* plants 3 days after herbicide treatment. Untreated plants were sprayed with water (Control; C). Plants were treated with 0.21 kg ha^−1^ of glyphosate (0.25 G), 0.84 kg ha^−1^ of glyphosate (1 G), 1.5 mg L^−1^ of imazamox (I) or their mixtures (0.25 G + I and 1 G + I). (Mean ± SE; n = 4). In each graph, different letters refer to statistically significant differences between treatments (two-way ANOVA followed by the Bonferroni *post hoc* test (P < 0.05)).

The effect of treatments on the most significant parameters of the free amino acid profile of was evaluated (Fig. 6). The general increase in the content of free amino acids could mask the specific changes in each absolute value of AAA^11^; therefore, the relative contents of AAA and BCAA are represented in terms of percentage of total free amino acid (Fig. 6A,B) in response to all treatments. In the GS population, the relative AAA content (Fig. 6A) increased after both doses of glyphosate, it was not affected by imazamox and mixtures had a similar or less effect than glyphosate. In GR plants, no changes were viewed in terms of relative AAA content (Fig. 6A). In the GS population, there was a significant increase of relative BCAA content after both doses of glyphosate compared to control, which was not observed in the GR population (Fig. 6B). A decrease in the relative content of BCAA after imazamox treatment was detected in both populations (Fig. 6B) and, mixtures had a similar or less effect than glyphosate alone.

In the GS population, both glyphosate doses produced a significant increase in total free amino acid content with respect to control, while no free amino acid accumulation was detected in the GR population after any of the glyphosate doses (Fig. 6C). Imazamox alone induced free amino acid accumulation in both populations. In the GS population, the level of free amino acid accumulation was the same for the mixtures and for the individual herbicide applications, while in the GR population, the level was lower for the mixtures than for imazamox alone. The relative content of acidic amino acids displayed a general decrease response in both populations after glyphosate and after imazamox in GR (Fig. 6D). Mixtures had a similar (in the case of GS) or less effect (in the case of GR) than glyphosate or imazamox alone. It was detected a general and significant increase in the relative content of amide amino acids after all treatments in GS population (Fig. 6E). In GR population, only 1G and I provoked a significant increase of amide amino acid content, that was similar or higher than the increase detected after mixtures (Fig. 6E).

Glyphosate treatment resulted in an increase of shikimate content in the GS population at levels 5-fold greater than in the GR population (Fig. 7A), confirming the resistance to glyphosate of GR plants. Imazamox applied alone did not modify shikimate content in any of the populations and in the GR population, shikimate accumulation was similar after herbicide mixtures application of glyphosate and imazamox and after glyphosate alone, as could be expected because shikimate is not directly related to the BCAA biosynthetic pathway. Surprisingly, significantly less shikimate accumulation was detected in the GS population when 0.25 G glyphosate treatment was applied with imazamox (Fig. 7A), compared to 0.25 G alone (about 82% lower).

TSS (the sum of glucose, fructose, and sucrose) and starch contents were measured in the leaves of both populations (Fig. 7B,C). It was observed a general increase in TSS and starch content in the GS population after glyphosate and after imazamox in the GR population. There were no significant differences on TSS or starch content between glyphosate and imazamox applied individually or as mixtures in any of the populations.

## Discussion

### Physiological effects of treatments in *A. palmeri* plants

Shikimate content is a well-known stress marker: it accumulates when the EPSPS enzyme is inhibited by glyphosate^13,46,51–58^. We observed that shikimate accumulated more in sensitive than in resistant plants (Fig. 7A). The mixture of imazamox with 0.25 G alleviated the shikimate accumulation induced by glyphosate alone.

An increase in the protein turnover rate after herbicide treatment^59^ was proposed to explain the increase in the size of the free amino acid pool and the general decrease in total soluble proteins induced by amino acid biosynthesis-inhibiting herbicides. Isotopic studies in *A. palmeri* revealed that both *de novo* synthesis of amino acids and protein turnover contribute to AAA accumulation in response to glyphosate^16^. Total free amino acid content was determined as a physiological marker of the effect of the herbicides (Fig. 6C). In the GS population, both glyphosate doses produced a significant increase in total free AA content with respect to control, as it has been reported before^11,13,15,17,18,60–62^. Due to the resistance to glyphosate, a smaller amount of free amino acids accumulated in the GR population after glyphosate treatment. Imazamox alone induced free amino acid accumulation in both populations, as has been previously reported^17^. In the GS population, the amounts of free amino acid accumulation were the same after the application of the mixtures and after individual herbicide application and in the GR population, the mixtures yielded lower amounts of free amino acids, evidencing that the effect was not exacerbated when herbicides were applied together.

The contents of two other groups of amino acids with known behaviors (acidic amino acids (Glu and Asp) and amide amino acids (Gln and Asn))^13,15^, were also determined after treatment with amino acid biosynthesis inhibitors (Fig. 5D,E). The general and significant decreases in acidic amino acid contents and the significant increases in amide amino acid contents detected in both populations had been previously reported^13,15^. In both groups of amino acids the mixtures induced less changes than herbicides alone, evidencing that with mixture application, the effect was the same or less than when herbicides were applied alone.

AAA accumulation after glyphosate treatment was reported as a physiological marker of damage^11,13,15,46^, thus the increase of AAA in the GS population and no in the GR population (Fig. 5A) suggests a higher level of damage on the GS due to its higher sensitivity to the herbicide. As AAA accumulation after glyphosate treatment implies higher damage, the maintenance of AAA levels with glyphosate and mixtures indicates that the effect is not exacerbated due to the presence of both herbicides. On the other hand, the dose of glyphosate treatment was too low to see a significant increase in relative BCAA content in the GR population, but in the GS population, there was a significant increase in the BCAA level with both doses of glyphosate compared to control (Fig. 5B), which is consistent with previous reports^13,63^ and with the AAA results (Fig. 5A). Interestingly, it was possible to detect a decrease in the relative content of BCAA after imazamox treatment in both populations (Fig. 7B). Previous studies reported transient decreases in the proportion of amino acids whose pathways were specifically inhibited by AHAS-inhibitors^13,64,65^.

Carbohydrates accumulate in response to the application of glyphosate^11,12,15,18^ and AHAS-inhibitors^14,15,49^, which can be a physiological marker of herbicide toxicity. Total soluble sugars and starch contents were measured in the leaves of both populations (Fig. 7B,C). The carbohydrate accumulation detected in leaves after herbicide treatment has been previously attributed to growth arrest. The accumulation of unused carbohydrates in sinks abolishes the sugar gradient required for long-distance transport, and carbohydrates accumulate in the leaves of treated plants because of a decrease in sink strength^18^. If carbohydrates are used as a physiological marker of stress, then the absence of differences in carbohydrate accumulation between mixtures and individual herbicides could indicate that the application of both herbicides does not increase the toxicity of the herbicides applied alone.

AHAS inhibition by imazamox induced free amino acid and carbohydrate accumulation in the leaves of *A. palmeri* (Figs. 6 and 7). Collectively, these results indicate that both populations show typical physiological markers as toxic consequences of BCAA biosynthesis-inhibiting herbicides which have been reported previously in other species^15,17^. Interestingly, such physiological effects were detected in both populations, supporting that *EPSPS* overexpression does not affect the physiological effects of AHAS inhibitors.

### Coordinated expression response was not detected after AHAS inhibition

The previously described general induction in the expression of all the genes of the AAA pathway, with the exception of *CM*, after glyphosate^46^, was not observed in the GS and GR populations probably due to the low doses applied. The expression pattern of the genes of the BCAA pathway was also evaluated (Fig. 2). Both of the populations generally showed no significant change in gene expression after glyphosate treatment, as previously reported^46^. No significant changes in gene expression were detected after the inhibition of one of the enzymes of the pathway, AHAS, by imazamox alone, as recently described for *Echinochoa colona*^66^. It was previously reported that the BCAA enzyme transcript levels increased after treatment with pyroxsulam^67^ (another AHAS inhibitor) within a short period of time (less than 48 hours). However, in agreement with our results, the slight change of BCAA enzyme gene expression level induced by another AHAS-inhibitor (imazapyr) provided evidence that transcriptional regulation may not be a major regulatory mechanism of the synthesis of BCAA^68^. Herbicide mixtures did not induce any remarkable change in the relative expression of the genes in this pathway (Fig. 2, Supplementary Table 1).

A similar regulatory program, combining transcriptional and posttranslational controls in response to abiotic stresses, was proposed to the metabolic pathways of 11 amino acids, including AAA and BCAA biosynthesis^69^. Specifically, it was proposed that allosteric biosynthetic enzymes respond post-translationally to changes in the level of the amino acids^69^. Nevertheless, our results show an opposite transcriptional behavior between the BCAA and AAA pathways. The inactivation of the BCAA biosynthetic pathway at the level of AHAS by imazamox did not provoke any significant or common pattern in the relative expression level of the other genes of the same pathway. In contrast, after glyphosate treatment, it has been described a general increase in the transcript levels of the enzymes of the shikimate pathway. This different effect after imazamox or glyphosate cannot be explained by any specific pattern in the content of amino acids whose specific biosynthesis is inhibited because decreases, increases and no changes have all been reported depending on the population.

As previously reported^46^, glyphosate did not provoke any significant change in the transcriptional levels of the genes of the BCAA pathway. The lack of effect of imazamox on the transcript levels of the genes of the AAA pathway confirms that no cross regulation exists between AAA and BCAA pathways, in spite of similar patterns in the content of free amino acids and carbohydrates after imazamox or glyphosate treatment.

To evaluate if the changes in gene transcription of the enzymes of the AAA pathway were reflected by the protein levels, EPSPS and DAHPS enzyme amounts were studied (Fig. 4). The importance of the DAHPS protein is based on its control of the entrance of carbon flux to AAA pathway^69^. In the GS population, only in the 1 G + I treatment was a significant increase of the protein detected with respect to imazamox and in the GR; there were no significant differences between treatments (Fig. 4B), as has been reported previously^46^. The EPSPS protein content in the absence of herbicides was 10-fold higher in *A. palmeri* GR population than in GS (Fig. 5B) due to the amplification of *EPSPS*, as has been previously reported^11,29,46,57,70^. There were non-significant increases in the EPSPS protein levels in the GS and GR populations after treatments (Fig. 5D), as previously reported for low doses of glyphosate^11,46^. The induction of DAHPS and EPSPS gene expression after 1 G + I treatment in the GS population and DHAPS gene expression after 1 G in the GR population (Fig. 3A–C) had no clear relation with DAHPS or EPSPS protein levels (Fig. 3B–D), which suggests the presence of post-transcriptional factors added to the transcriptional regulation, as has been suggested before^46^.

### In both populations, joint application of the herbicides did not exacerbate the physiological effects provoked by the herbicides alone

In general, the changes detected in amino acid and carbohydrate contents in response to herbicide mixtures were similar to the changes detected after individual treatments and less than the sum of the individual effects; evidencing that physiological perturbations were not exacerbated if both herbicides were applied together. The detected increase in carbohydrates and free amino acid pool were maximum with the application of one herbicide alone, and were not increased with the presence of the other, suggesting that the effect of each individual herbicide was maximum and that there was a common pattern in amino acid and carbohydrate content after imazamox or glyphosate application.

Both populations behaved similar after mixture treatments, evidencing that in this resistant population it was independent of the *EPSPS* copy number, as it was detected regardless of sensitivity or resistance to glyphosate. It should be pointed out that this study focuses only on one population and other physiological behaviors might be present in other glyphosate-resistant biotypes. Additionally, the comparison of *A. palmeri* sensitive populations with AHAS resistant populations or with multiple resistant populations to glyphosate and AHAS inhibitors, which are a growing worldwide problem^41^, would be very valuable to get new insights in the evaluation of the weed physiological performance after glyphosate and/or AHAS inhibitors.

There is one exception in that similar behavior of both populations: a clearly different pattern was detected at the transcriptional level in AAA pathway enzymes in the GS population compared to the GR population when they were treated with mixtures. The GS population showed an enhancement effect after mixtures and the GR population showed a strong alleviation that even brought down transcript levels below control levels in the mixtures. This particular transcriptional pattern detected in GR plants could be related to *EPSPS* gene amplification and overexpression. Further investigations to clarify the molecular mechanism underlying this specific transcriptional response of GR would be very useful.

### Physiological antagonism might limit the agronomic use of glyphosate and AHAS mixtures

Herbicide mixtures can interact in three different ways: antagonistically, additively, and synergistically^40^. Although some additive results have been reported before^36,38,39^, antagonism between AHAS-inhibitors (specifically imidazolinones) and glyphosate has been previously reported in dose-response studies^34,35,37^, although their effect in physiological parameters was not tested. As both herbicides have been described to have some common physiological effects, joint application of glyphosate and AHAS-inhibitors may cause noteworthy changes (alleviation or enhancement) in the physiological effects elicited by the herbicides applied alone. In this study, physiological parameters have been used as indicators of additivity, synergism and antagonism. As in both populations the mixture effects were mostly less than the sum of the individual effects, antagonism was the general behavior detected in all parameters.

In conclusion, and considering the general pattern, it can be suggested that in this population of *A. palmeri*, glyphosate and imazamox applied together are physiologically antagonistic; the evidence for this conclusion is based on finding toxicity markers that are affected in the mixture to the same extent or even less than with one herbicide acting alone. This behavior would have practical implications that would implicate changes in the agronomic use of the mixtures of these herbicides. On one hand, when both herbicides are applied together the recommended field rates of both herbicides have to be fully applied as toxic effects are not additive, and the application rates cannot be reduced. On the other hand, as with mixtures two different target sites are inhibited, it could be used to control resistant populations to one type of herbicides. Nevertheless, mixture treatments have to be used carefully as they will increase the risk of selecting multiple resistant populations to both types of herbicides.

## Supplementary information

Supplemental Tables 1-4 and Figs. 1-3
