# Supplementary material for: Physiological performance of glyphosate and imazamox mixtures on Amaranthus palmeri sensitive and resistant to glyphosate
Source: Sci Rep. 2019 Dec 3;9:18225. doi: 10.1038/s41598-019-54642-9 (PMC6890711; doi:10.1038/s41598-019-54642-9)
Supplement: Supplementary file 1 — Supplemental Tables 1-4 and Figs. 1-3 [file 41598_2019_54642_MOESM1_ESM.pdf]

<u>SUPPLEMENTARY DATA</u>

Physiological performance of glyphosate and imazamox mixtures on *Amaranthus palmeri* sensitive and resistant to glyphosate

Manuel Fernández-Escalada, Ainhoa Zulet-González, Miriam Gil-Monreal, Mercedes Royuela, Ana Zabalza

Supplementary Table 1. Results of the two-way analysis of variance for all measured parameters in the **sensitive population** (GS) and **resistant** population (GR). Significance level $p < 0.05$ (* = $p < 0.05$; ns = not significant). Transcript abundance in genes of the enzymes of the aromatic amino acid (AAA) pathway: D-arabino- heptulosonate 7-phosphate synthase (DAHPS); dehydroquinate synthase (DHQS); 3-dehydroquinate dehydratase/shikimate dehydrogenase (DQSD); shikimate kinase (SK); 5-enolpyruvylshikimate 3-phosphate synthase (EPSPS); chorismate synthase (CS); chorismate mutase (CM) and anthranilate synthase (AS). Enzymes of the branched-chain amino acid (BCAA) pathway: acetohydroxy acid synthase (AHAS); acetohydroxyacid isomer reductase (AHAIR); dihydroxyacid dehydratase (DHAD) and BCAA transaminase (TA). DAHPS and EPSPS protein content. AAA content: tryptophan (Trp), tyrosine (Tyr), phenylalanine (Phe) and AAA percentage of the total free amino acid pool. BCAA content: leucine (Leu), valine (Val), isoleucine (Ile) and BCAA percentage of the total free amino acid. Total free amino acid content, acidic amino acids (glutamic acid (Glu) + aspartate (Asp)) and amide amino acids (glutamine (Gln) + asparagine (Asn)) as percentage of the total free amino acid pool. Total soluble sugar (fructose. glucose and sucrose) (TSS) and starch content.

	**Sensitive population (GS)**			**Resistant population (GR)**		
**Parameter**	**Imazamox**	**Glyphosate**	**Interaction (imazamox x glyphosate)**	**Imazamox**	**Glyphosate**	**Interaction (imazamox x glyphosate)**
DAHPS expr.	*	*	ns	*	ns	*
DHQS expr.	*	*	ns	*	*	*
DQSD expr.	*	ns	ns	*	*	*
SK expr.	*	*	*	*	*	ns
EPSPS expr.	*	*	*	ns	ns	*
CS expr.	*	*	ns	ns	*	*
CM expr.	ns	*	ns	*	ns	*
AS expr.	*	*	ns	ns	*	*
AHAS expr.	*	ns	ns	ns	*	*
AHAIR expr.	*	ns	ns	ns	*	ns
DHAD expr.	ns	ns	ns	*	*	*
TA expr.	*	*	ns	ns	ns	ns
DHAPS protein	ns	*	ns	ns	ns	ns
EPSPS protein	ns	*	ns	*	*	ns
Shikimate	*	*	*	ns	*	*
Trp	ns	*	ns	*	*	*
Tyr	ns	*	ns	*	*	*
Phe	ns	*	ns	*	*	*
Leu	*	*	ns	ns	*	ns
Val	*	*	ns	ns	*	*
Ile	*	*	ns	*	*	ns
%AAA	ns	*	ns	ns	ns	*
%BCAA	*	*	ns	ns	*	*
%Acidic	ns	*	ns	*	*	*
%Amide	*	*	*	*	*	*
TotalAA	ns	*	*	*	*	*
TSS	ns	*	*	*	ns	ns
Starch	*	*	*	*	ns	*

Supplementary Table 2. Transcript abundance in genes of the enzymes of the aromatic amino acid pathway: D-arabino- heptulosonate 7-phosphate synthase (DAHPS); dehydroquinate synthase (DHQS); 3-dehydroquinate dehydratase/shikimate dehydrogenase (DQSD); shikimate kinase (SK); 5-enolpyruvylshikimate 3-phosphate synthase (EPSPS); chorismate synthase (CS); chorismate mutase (CM) and anthranilate synthase (AS) and enzymes of the branched-chain amino acid pathway: acetohydroxy acid synthase (AHAS); acetohydroxyacid isomer reductase (AHAIR); dihydroxyacid dehydratase (DHAD) and BCAA transaminase (TA). Relative transcript abundance was normalized using the normalization gene beta tubulin and each population to its own control in *Amaranthus palmeri* plants 3 days after herbicide treatment in sensitive (**GS**) and resistant (**GR**) populations. Untreated plants were sprayed with water (Control; C). Plants were treated with 0.21 kg ha^{-1} of glyphosate (0.25G). 0.84 kg ha^{-1} of glyphosate (1G). 1.5 mg L^{-1} of imazamox (I) or their mixtures (0.25G+I and 1G+I). (Mean ± SE; n=4). Different letters in a row refer to statistically significant differences between treatments (two-way ANOVA followed by the Bonferroni *post hoc* test (P<0.05).

**GS**	**CONTROL**	**0.25 G**	**1 G**	**I**	**0.25G+I**	**1G+I**
**DAHPS**	1.04 ± 0.15 b	5.54 ± 1.41 ab	6.49 ± 3.19 ab	4.48 ± 1.40 ab	8.86 ± 1.65 ab	12.58 ± 3.45 a
**DHQS**	1.08 ± 0.23 c	5.09 ± 1.70 bc	2.80 ± 0.43 bc	3.31 ± 0.56 bc	8.52 ± 0.95 ab	12.05 ± 2.46 a
**DQSD**	1.06 ± 0.18 a	3.81 ± 0.74 a	1.40 ± 0.08 a	2.92 ± 0.49 a	5.06 ± 1.29 a	6.08 ± 2.84 a
**SK**	1.04 ± 0.16 c	1.83 ± 0.53 c	3.88 ± 0.52 b	2.04 ± 0.45 c	5.20 ± 0.55 ab	5.78 ± 0.37 a
**EPSPS**	1.03 ± 0.15 c	2.62 ± 0.83 c	3.46 ± 0.26 bc	2.73 ± 0.67 c	6.49 ± 1.20 ab	9.33 ± 1.01 a
**CS**	1.01 ± 0.06 b	1.84 ± 0.37 ab	2.62 ± 0.77 ab	2.20 ± 0.23 ab	3.95 ± 0.34 ab	5.27 ± 1.72 a
**CM**	1.01 ± 0.12 ab	0.36 ± 0.07 b	0.59 ± 0.13 ab	1.17 ± 0.39 a	0.54 ± 0.15 ab	0.89 ± 0.14 ab
**AS**	1.14 ± 0.34 c	9.33 ± 3.94 abc	8.12 ± 2.26 abc	8.10 ± 2.17 abc	19.43 ± 3.46 a	16.63 ± 2.54 ab
**AHAS**	1.08 ± 0.23 a	1.04 ± 0.11 a	1.53 ± 0.71 a	3.08 ± 0.76 a	2.28 ± 0.45 a	3.11 ± 1.29 a
**AHAIR**	1.02 ± 0.12 a	1.12 ± 0.12 a	1.22 ± 0.39 a	2.17 ± 0.40 a	1.93 ± 1.19 a	1.74 ± 0.35 a
**DHAD**	1.03 ± 0.15 a	0.64 ± 0.18 a	0.97 ± 0.28 a	1.45 ± 0.24 a	0.95 ± 0.19 a	1.37 ± 0.35 a
**TA**	1.02 ± 0.12 b	2.74 ± 0.64 ab	2.67 ± 0.57 ab	3.37 ± 1.16 ab	4.51 ± 0.97a	4.65 ± 1.61 a

**GR**	**CONTROL**	**0.25 G**	**1 G**	**I**	**0.25G+I**	**1G+I**
**DAHPS**	1.03 ± 0.15 b	1.11 ± 0.67 b	2.67 ± 0.64 a	1.03 ± 0.16 b	1.24 ± 0.23 b	0.42 ± 0.04 b
**DHQS**	1.06 ± 0.24 b	1.11 ± 0.23b	7.08 ± 2.44 a	2.96 ± 0.62 b	1.45 ± 0.48 b	1.20 ± 0.31 b
**DQSD**	1.05 ± 0.21 a	1.16 ± 0.24 a	2.28 ± 0.94 a	2.97 ± 1.16 a	0.67 ± 0.09 a	0.72 ± 0.23 a
**SK**	1.00 ± 0.06 d	4.44 ± 0.07 c	7.34 ± 1.03 a	4.66 ± 1.32 bc	7.28 ± 0.77 a	5.21 ± 0.21 ab
**EPSPS**	1.10 ± 0.32 a	2.47 ± 1.82 a	3.21 ± 1.48 a	2.60 ± 0.91 a	1.91 ± 0.53 a	1.09 ± 0.24 a
**CS**	1.00 ± 0.07 b	0.88 ± 0.09 b	4.02 ± 1.73 a	1.22 ± 0.20 b	1.69 ± 0.13 b	0.96 ± 0.20 b
**CM**	1.01 ± 0.10 a	0.45 ± 0.07 b	1.12 ± 0.15 a	0.55 ± 0.10 ab	0.60 ± 0.30 ab	0.24 ± 0.12 b
**AS**	1.00 ± 0.03 a	2.46 ± 1.02 a	4.38 ± 1.95 a	2.37 ± 0.57 a	4.44 ± 0.57 a	2.49 ± 0.37 a
**AHAS**	1.03 ± 0.18 b	1.32 ± 0.49 b	3.27 ± 1.00 a	1.30 ± 0.13 b	1.17 ± 0.37 b	1.49 ± 0.52 b
**AHAIR**	1.26 ± 0.76 b	5.24 ± 0.78 a	4.27 ± 1.05 ab	3.51 ± 1.88 ab	3.99 ± 0.28 ab	3.31 ± 0.70 ab
**DHAD**	1.04 ± 0.21 ab	0.69 ± 0.12 b	1.97 ± 0.87 a	0.76 ± 0.19 ab	0.74 ± 0.04 ab	0.60 ± 0.05 ab
**TA**	1.05 ± 0.23 a	1.71 ± 0.62 a	2.09 ± 0.25 a	2.26 ± 0.50 a	1.46 ± 0.20 a	1.92 ± 0.47 a

Supplementary Table 3. Shikimate content. Aromatic amino acid content (AAA) content: tryptophan (Trp), tyrosine (Tyr), phenylalanine (Phe) and AAA percentage of the total free amino acid pool. Branched chain amino acid (BCAA) content: leucine (Leu), valine (Val), isoleucine (Ile) and BCAA percentage of the total free amino acid. Total free amino acid content, acidic amino acids (glutamic acid (Glu) + aspartate (Asp)) and amide amino acids (glutamine (Gln) + asparagine (Asn)) as percentage of the total free amino acid pool. Total soluble sugar (fructose. glucose and sucrose) (TSS) and starch contents. Shikimate, individual amino acids, total amino acid and carbohydrate contents are expressed in µg disc^{-1}, nmol g^{-1} FW, µmol g^{-1} FW or mg g^{-1} FW, respectively. Samples were taken 3 days after herbicide treatment in sensitive (**GS**) *Amaranthus palmeri* population. Untreated plants were sprayed with water (Control; C). Plants were treated with 0.21 kg ha^{-1} of glyphosate (0.25G), 0.84 kg ha^{-1} of glyphosate (1G), 1.5 mg L^{-1} of imazamox (I) or their mixtures (0.25G+I and 1G+I). (Mean ± SE; n=4). Different letters in a row refer to statistically significant differences between treatments (two-way ANOVA followed by the Bonferroni *post hoc* test (p<0.05).

GS	CONTROL	0.25 G	1 G	I	0.25G+I	1G+I
**SHIKIMATE**	0.290 ± 0.040 b	7.025 ± 1.527 a	10.382 ± 0.998 a	0.291 ± 0.044 b	1.223 ± 0.577 b	9.779 ± 1.984 a
**TRP**	26.02 ± 12.32 a	45.70 ± 2.25 a	38.80 ± 10.95 a	19.63 ± 4.82 a	33.67 ± 17.52 a	59.22 ± 19.95 a
**TYR**	12.20 ± 2.14 a	109.42 ± 14.05 a	97.52 ± 41.18 a	28.84 ± 3.22 a	79.01 ± 22.66 a	68.55 ± 14.70 a
**PHE**	55.36 ± 6.77 b	368.12 ± 31.15 ab	457.09 ± 178.90 a	80.99 ± 11.13 b	296.71± 79.65 ab	325.64 ± 67.41 ab
**LEU**	137.86 ± 9.25 bc	382.43 ± 41.97 ab	453.48 ± 143.91 a	68.23 ± 12.95 c	190.15 ± 37.42 abc	360.59 ± 80.08 ab
**VAL**	141.28 ± 6.82 b	728.00 ± 81.41 a	683.48 ± 159.76 a	97.08 ± 39.69 b	286.16 ± 86.13 ab	542.80 ± 189.32 ab
**ILE**	81.19 ± 4.99 d	444.00 ± 20.07 ab	510.47 ± 35.38 a	115.97 ± 6.35 cd	271.55 ± 43.91 bc	420.68 ± 92.70 ab
**AAA**	0.46 ± 0.10 bc	1.50 ± 0.12 a	1.48 ± 0.48 a	0.39 ± 0.05 c	1.29 ± 0.18 ab	1.21 ± 0.27 abc
**BCAA**	2.05 ± 0.11 b	4.16 ± 0.23 a	4.60 ± 0.61 a	0.86 ± 0.18 c	2.27 ± 0.46 b	3.20 ± 0.39 ab
**ACID**	50.13 ± 1.31 a	22.37 ± 2.32 b	22.72 ± 2.29 b	33.14 ± 4.94 ab	22.60 ± 1.62 b	21.48 ± 5.07 b
**AMIDE**	7.56 ± 0.39 b	36.85 ± 1.06 a	36.03 ± 2.36 a	29.23 ± 6.80 a	33.79 ± 2.04 a	41.83 ± 7.01 a
**TOTALAA**	17.68 ± 0.90 b	46.01 ± 8.86 a	37.07 ± 2.47 a	32.89 ± 1.08 a	36.85 ± 4.24 a	39.35 ± 5.80 a
**TSS**	0.68 ± 0.04 b	5.96 ± 0.55 a	4.43 ± 0.15 a	4.07 ± 1.84 ab	6.03 ± 0.92 a	5.29 ± 0.73 a
**STARCH**	69.15 ± 25.01 b	469.56 ± 91.70 a	628.88 ± 210.31 a	511.03 ± 119.63 ab	660.24 ± 166.63 a	446.49 ± 187.13 ab

Supplementary Table 4. Shikimate content. Aromatic amino acid content (AAA) content: tryptophan (Trp), tyrosine (Tyr), phenylalanine (Phe) and AAA percentage of the total free amino acid pool. Branched chain amino acid (BCAA) content: leucine (Leu), valine (Val), isoleucine (Ile) and BCAA percentage of the total free amino acid. Total free amino acid content, acidic amino acids (glutamic acid (Glu) + aspartate (Asp)) and amide amino acids (glutamine (Gln) + asparagine (Asn)) as percentage of the total free amino acid pool. Total soluble sugar (fructose. glucose and sucrose) (TSS) and starch contents. Shikimate, individual amino acids, total amino acid and carbohydrate contents are expressed in μg $disc^{-1}$, nmol g^{-1} FW, μmol g^{-1} FW or mg g^{-1} FW, respectively. Samples were taken 3 days after herbicide treatment in resistant (**GR**) *Amaranthus palmeri* population. Untreated plants were sprayed with water (Control; C). Plants were treated with 0.21 kg ha^{-1} of glyphosate (0.25G), 0.84 kg ha^{-1} of glyphosate (1G), 1.5 mg L^{-1} of imazamox (I) or their mixtures (0.25G+I and 1G+I). (Mean ± SE; n=4). Different letters in a row refer to statistically significant differences between treatments (two-way ANOVA followed by the Bonferroni *post hoc* test (p<0.05).

GR	CONTROL	0.25 G	1 G	I	0.25G+I	1G+I
**SHIKIMATE**	0.38 ± 0.05 b	0.71 ± 0.43 ab	2.04 ± 0.78 a	0.35 ± 0.05 b	0.42 ± 0.10 b	1.29 ± 0.62 ab
**TRP**	25.54 ± 3.52 c	28.78 ± 9.91 bc	19.74 ± 6.78 c	86.29 ± 9.03 a	61.72 ± 13.56 ab	26.74 ± 3.88 bc
**TYR**	12.46 ± 1.42 c	44.18 ± 8.38 a	20.41 ± 5.71 bc	52.61 ± 3.10 a	40.78 ± 6.13 ac	15.45 ± 3.67 c
**PHE**	38.43 ± 10.53 c	176.35 ± 34.75 ab	92.52 ± 19.99 bc	234.29 ± 14.81 a	148.53 ± 20.19 ab	95.35 ± 17.32 bc
**LEU**	99,77 ± 14,38 b	62,52 ± 14,17 b	135,83 ± 33,33 ab	66,67 ± 10,84 b	84,94 ± 17,05 b	200,99 ± 22,64 ab
**VAL**	123.00 ± 15.97 bc	84.33 ± 7.15 cd	191.90 ± 23.47 ab	34.84 ± 6.06 d	106.34 ± 2.71 bcd	254.28 ± 22.93 a
**ILE**	92.81 ± 14.61 b	203.46 ± 40.36 ab	195.26 ± 48.12 ab	277.55 ± 63.50 a	366.24 ± 28.50 a	210.95 ± 26.92 ab
**AAA**	0.41 ± 0.03 a	0.79 ± 0.10 a	0.48 ± 0.14 a	0.67 ± 0.05 a	0.54 ± 0.08 a	0.61 ± 0.14 a
**BCAA**	1.70 ± 0.21 ab	1.33 ± 0.15 b	1.92 ± 0.39 ab	0.65 ± 0.12 c	1.34 ± 0.00 b	2.51 ± 0.03 a
**ACIDIC**	48.39 ± 1.36 a	37.57 ± 1.70 b	34.65 ± 2.94 b	14.53 ± 0.71 d	20.84 ± 1.09 c	41.12 ± 1.54 ab
**AMIDE**	12.35 ± 2.55 c	17.27 ± 0.68 bc	30.42 ± 5.07 b	60.80 ± 1.01 a	51.57 ± 1.82 a	18.44 ± 2.73 bc
**TOTALAA**	18.47 ± 0.62 d	30.37 ± 0.95 c	28.38 ± 3.24 cd	62.72 ± 3.98 a	47.00 ± 1.36 b	21.35 ± 1.27 cd
**TSS**	0.66 ± 0.08 b	3.20 ± 0.48 ab	2.97 ± 1.02 ab	5.47 ± 0.43 a	6.35 ± 0.77 a	4.30 ± 1.22 ab
**STARCH**	94.55 ± 58.81 b	640.39 ± 129.73 ab	422.37 ± 154.11 ab	1017.62 ± 193.60 a	651.15 ± 110.87 ab	572.50 ± 104.91 ab

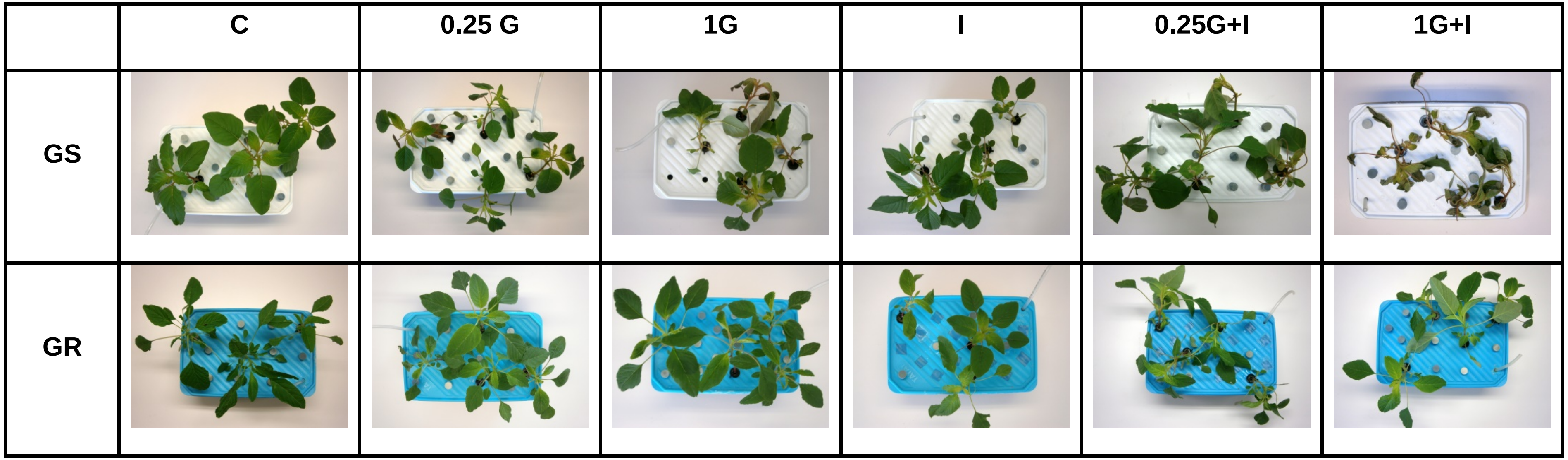

Supplementary Figure 1. Aspect of glyphosate-sensitive (GS) and glyphosate-resistant (GR) *A. palmeri* 3 days after treatment. Untreated plants were sprayed with water (Control; C). Plants were treated with 0.21 kg ha^{-1} of glyphosate (0.25G), 0.84 kg ha^{-1} of glyphosate (1G), 1.5 mg L^{-1} of imazamox (I) or their mixtures (0.25G+I and 1G+I).

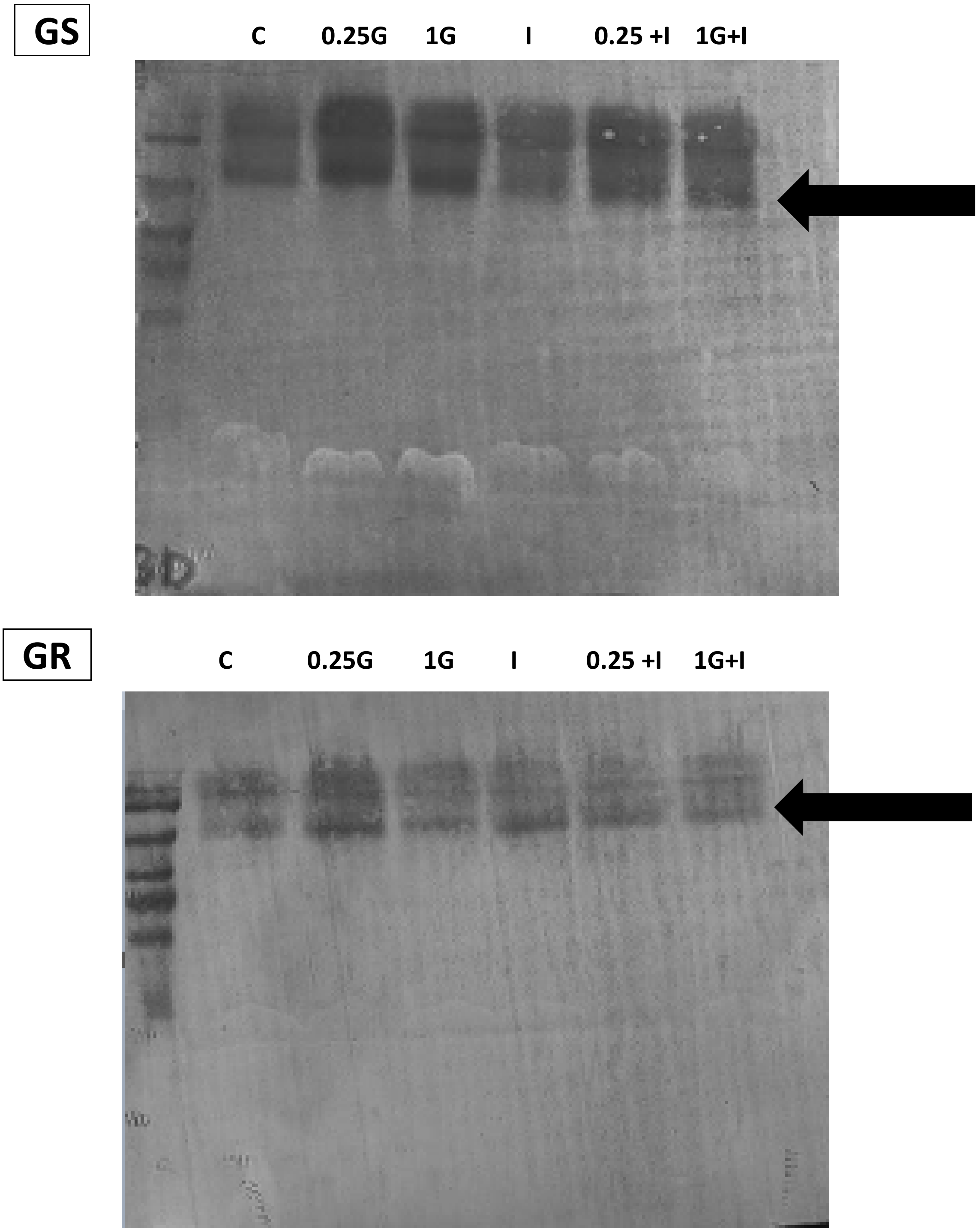

Supplementary Figure 2. Representative DAHPS immunoblots of glyphosate-sensitive and glyphosate-resistant (GR) populations. Total soluble protein (40 μg per well) were fractioned by 12.5% SDS-PAGE and blotted. Untreated plants were sprayed with water (Control; C). Plants were treated with 0.21 kg ha^{-1} of glyphosate (0.25G), 0.84 kg ha^{-1} of glyphosate (1G), 1.5 mg L^{-1} of imazamox (I) or their mixtures (0.25G+I and 1G+I). Black arrows indicate the position of DAHPS protein.

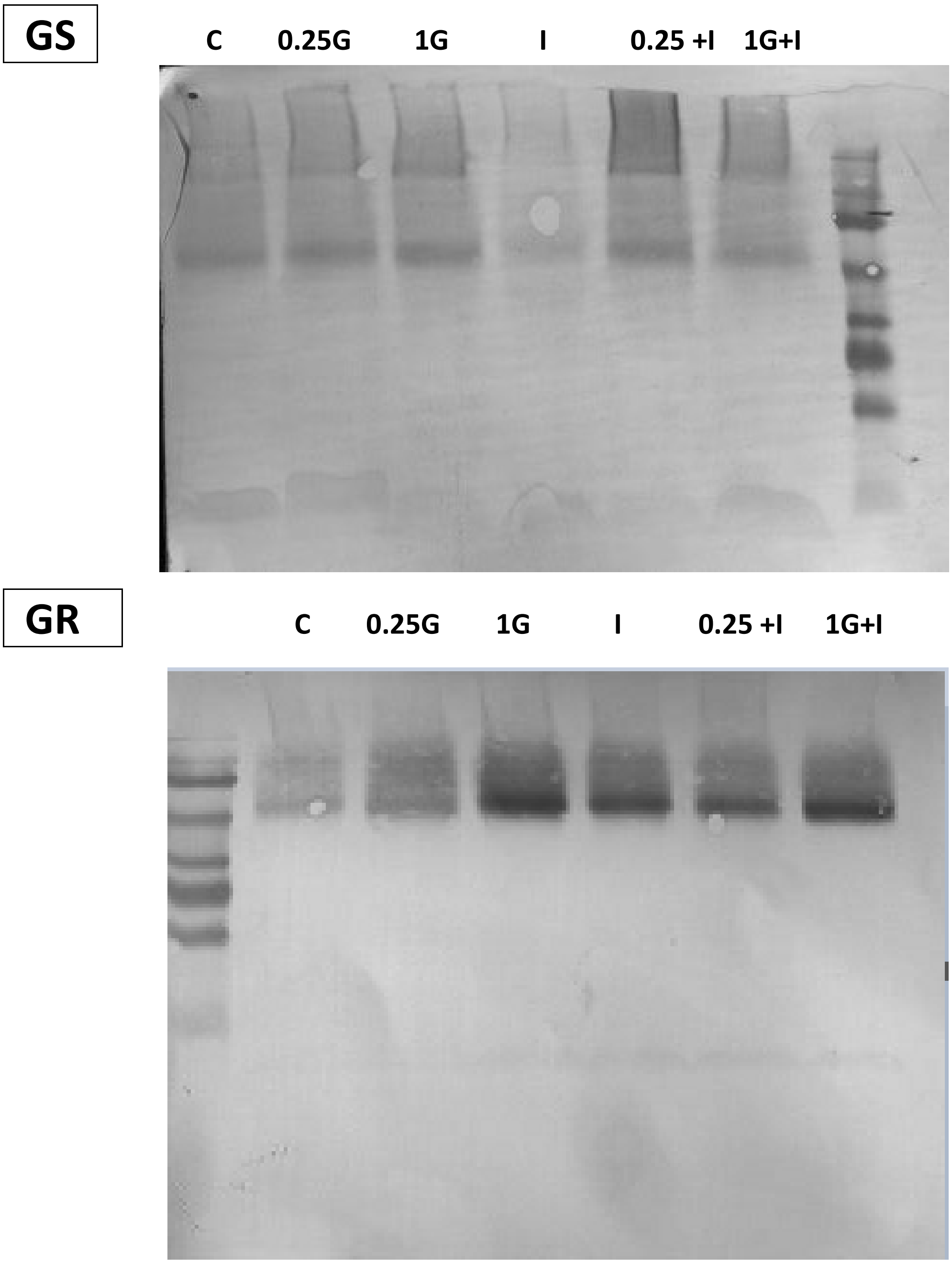

Supplementary Figure 3. Representative EPSPS immunoblots of glyphosate-sensitive and glyphosate-resistant (GR) populations. Total soluble protein (80 μg per well in GS and 15 μg per well in GR) were fractioned by 12.5% SDS-PAGE and blotted. Untreated plants were sprayed with water (Control; C). Plants were treated with 0.21 kg ha^{-1} of glyphosate (0.25G), 0.84 kg ha^{-1} of glyphosate (1G), 1.5 mg L^{-1} of imazamox (I) or their mixtures (0.25G+I and 1G+I).
